# New Appliances and Things Medical

**Published:** 1903-03-28

**Authors:** 


### NEW APPLIANCES AND THINGS MEDICAL.

[We shall be glad to receive at our Office, 28 & 29 Southampton Street, Strand, London, W.C., from the manufacturers, specimens of all new preparations and appliances which may be brought out from time to time.]

### LAXATINE.

(F. Williams and Co., 83 Upper Thames Street, E.C. London Depôt, Wilcox and Co., 49 Haymarket, London, S.W.)

Laxatine is an ingenious combination of castor oil and magnesia. It is a preparation which is quite tasteless and apparently reliable in action. It is only mildy aperient in action, and a somewhat large dose, *i.e.* a tablespoonful, must be administered to obtain a satisfactory effect. It will probably prove most useful for the treatment of constipation in children.

### CHOWRY MUTHU'S INHALER FOR FORMALDEHYDE INHALATION.

(S. Maw, Son and Sons, 7–12 Aldersgate Street, London, E.C.)

This inhaler is pyramidal in shape and made of perforated zinc the edges of which are bound with black velvet; by means of elastic loops it is held in position over the mouth and nose with comparative comfort to the wearer. The constant inhalation of formaldehyde is regarded by those experienced in the method as one of the most efficacious means of treating pulmonary tuberculosis. The successful application of this method demands the use of an inhaler or mask which can be worn with comfort during the night and for the greater part of the day. The Chowry Muthu Inhaler appears to fulfil these conditions most satisfactorily.

### SANATOGEN.

(F. Williams and Co., 83 Upper Thames Street, London, E.C.)

Sanatogen is a desiccated food product containing 95 per cent. of casein, and 5 per cent. of glycero-phosphate of soda. It is tasteless and soluble in hot water and mineral waters containing carbonic acid gas in solution. Containing as it does the nutritive and proteid elements of milk in a concentrated form, and fortified by a nerve food such as glycerophosphate of soda, sanatogen constitutes a valuable food for the building up healthy tissue and for the repair of those weakened by debilitating disease. It is specially indicated for the treatment of rickets and neurasthenia.

